# Mechanistic insights into gut microbiota–driven autoimmunity in rheumatoid arthritis

**DOI:** 10.3389/fimmu.2026.1812972

**Published:** 2026-04-02

**Authors:** Jiawen Li, Xin Chen, Xingwen Xie

**Affiliations:** 1 Gansu University of Traditional Chinese Medicine, Lanzhou, China; 2 Affiliated Hospital of Gansu University of Traditional Chinese Medicine, Lanzhou, China

**Keywords:** autoimmunity, dysbiosis, gut microbiota, gut–joint axis, molecular mimicry, rheumatoid arthritis, short-chain fatty acids, Th17/Tregimbalance

## Abstract

Rheumatoid arthritis (RA) is a systemic autoimmune disease whose pathogenic drivers and initiating immune events remain incompletely understood. Increasing evidence implicates the gut–joint axis in RA, yet the mechanisms by which intestinal microbiota contribute to disease development still require integrative clarification. This review summarizes current experimental and clinical evidence on the role of gut dysbiosis in promoting autoimmunity in RA. We discuss alterations in microbial composition and their links to barrier dysfunction, immune-cell polarization, microbial metabolites, and antigen-specific immune responses. Human cohort studies and arthritis models suggest that reduced microbial diversity, loss of short-chain fatty acid (SCFA)-producing commensals, and expansion of taxa such as Prevotella copri and Collinsella are associated with impaired epithelial integrity, enhanced Th17/Tfh differentiation, reduced regulatory T- and B-cell activity, and increased autoantibody production. Mechanistic studies further support roles for molecular mimicry, microbially derived citrullinated antigens, and metabolite-mediated signaling in the breakdown of immune tolerance and persistence of synovial inflammation. We also discuss emerging microecology-based interventions, including probiotics, prebiotics, postbiotics, and fecal microbiota transplantation, together with their translational potential and current limitations. Overall, available evidence places gut microbiota-mediated immune remodeling at the center of RA pathogenesis and supports precision microbiome modulation as a promising adjunctive strategy for disease prevention and treatment.

## 1 Introduction

Rheumatoid arthritis (RA) is a chronic autoimmune disease characterized by synovial inflammation and progressive joint destruction, leading to substantial impairment in quality of life (1, 2). Its pathogenesis reflects a complex interplay between genetic susceptibility and environmental factors, although the precise etiologic drivers remain incompletely defined (3, 4). In recent years, the gut–joint axis has attracted increasing attention, with accumulating evidence suggesting that RA may have mucosal origins in which altered interactions between microbial communities and mucosal immunity trigger systemic autoimmune responses that ultimately damage the joints (5, 6). The gut is the body’s largest immune-associated organ and contains a complex microbial ecosystem whose structural and functional alterations may profoundly affect immune homeostasis (7, 8). A growing body of evidence indicates that gut dysbiosis is closely linked to the initiation and progression of RA (9, 10). In particular, perturbations of intestinal microbial ecology may contribute to the loss of immune tolerance and the onset of autoimmune processes during the early stages of RA (11, 12).

## 2 Roles of the Gut microecology in RA pathogenesis

### 2.1 Dysbiosis and mucosal barrier dysfunction

The dysbiotic microbial phenotype associated with RA is characterized by reduced gut microbial diversity, loss of beneficial taxa, and expansion of potentially harmful microorganisms (13, 14). Such dysbiosis can impair the integrity of the intestinal epithelial barrier, leading to increased intestinal permeability (“leaky gut”) and activation of mucosal immune responses, thereby promoting exposure to autoantigens and amplification of inflammation (15, 16). Patients with RA show evidence of subclinical intestinal inflammation and barrier dysfunction, including reduced expression of the tight-junction protein ZO-1 and increased zonulin levels in serum and feces, findings consistent with enhanced intestinal permeability (16, 17). In mouse models of collagen-induced arthritis (CIA) and rat models of adjuvant-induced arthritis (AIA), increased intestinal permeability and inflammatory infiltration can be detected before the onset of overt arthritis, supporting the concept that barrier disruption precedes joint disease (18). This process may be driven in part by elevated zonulin, and zonulin-targeted interventions have been shown to attenuate arthritis severity in mice (19, 20). Anti-inflammatory therapy in patients with active RA also reduces markers of intestinal permeability, suggesting that control of systemic inflammation may help restore barrier function (21). Together, these findings support the view that dysbiosis-associated barrier dysfunction is an important contributor to RA pathogenesis by facilitating translocation of bacteria and bacterial products into the lamina propria or systemic circulation, thereby promoting systemic immune activation (17, 22).

### 2.2 Regulation of immune equilibrium by the gut microbiota

Under homeostatic conditions, the gut microbiota coexists with the host immune system to maintain mucosal immune balance. In RA, however, dysbiosis can disrupt this balance and shift immune responses toward a pro-inflammatory state (7, 14). The importance of the microbiota in immune development is highlighted by germ-free animal studies, in which gut-associated lymphoid tissue is underdeveloped and effector T-cell populations, including Th17 cells, are markedly reduced (23). In arthritis-prone models, depletion of the intestinal microbiota through antibiotics or germ-free conditions substantially reduces Th17-cell frequencies and attenuates arthritis severity (24). Conversely, recolonization with conventional microbiota restores pathogenic Th17 responses and exacerbates disease (25). These findings indicate that the gut microbiota promotes pro-inflammatory Th17 responses and is a key determinant of autoimmune inflammation (17, 26).

By contrast, regulatory T (Treg) cells are central to the maintenance of immune tolerance, and commensal microbes can promote Treg induction. In particular, some Firmicutes are able to drive peripheral Treg differentiation and thereby suppress inappropriate immune activation (27). RA is often characterized by a reciprocal imbalance in which Treg abundance or function is reduced while pathogenic Th17 cells expand (28). Dysbiosis may promote Th17 polarization and suppress Treg responses through the combined effects of microbial metabolites and antigenic stimulation, thereby destabilizing Th17/Treg homeostasis and driving autoimmunity (28, 29). Importantly, microbiota-mediated immune regulation is not limited to T cells. Microbes and their components, such as lipopolysaccharide and peptidoglycan, can directly stimulate innate immune cells, including macrophages and dendritic cells, to produce pro-inflammatory cytokines such as IL-1 and TNF, thereby sustaining a chronic inflammatory milieu (22). Activated mucosal immune cells may subsequently circulate and home to synovial tissue, where they contribute to local inflammation (30). Collectively, disruption of gut microecology may impair immune tolerance through multiple pathways, skewing both innate and adaptive immunity toward pro-inflammatory programs and thereby providing an immunologic basis for the initiation and progression of RA (31, 32).

### 2.3 Systemic effects of microbial metabolites

The gut microbiota produces a wide range of metabolites that exert distal effects on host immunity and metabolism (33). Short-chain fatty acids (SCFAs), including acetate, propionate, and butyrate, are reduced in patients with RA, despite their important roles in immunomodulation (34–36). SCFAs promote the expansion of anti-inflammatory regulatory B and T cells, including IL-10+ B cells and Foxp3+ Treg cells, while suppressing excessive activation of pro-inflammatory effector T cells through inhibition of histone deacetylases and activation of G protein-coupled receptors, thereby contributing to immune homeostasis (37, 38). Among these metabolites, butyrate is particularly potent and is widely recognized as an effective histone deacetylase inhibitor; it enhances IL-10 production by B cells and promotes the development of follicular regulatory T (Tfr) cells, thereby restraining autoimmune responses (37). One of the characteristic features of the RA gut microbiota is a reduction in butyrate-producing microbes, including some Bacteroidaceae and Bacteroides, together with an increase in butyrate-consuming microbes, changes that may lower overall butyrate availability and diminish its protective effects (36).

The therapeutic potential of butyrate supplementation has also been supported by animal studies. Prophylactic administration of butyrate restores the balance between pathogenic Tfh cells and protective Tfr cells in CIA mice, reduces autoantibody production, and ameliorates arthritis (39). In another model, butyrate-induced metabolic regulation of B cells was essential for the anti-arthritic effect, because this protection was not observed in B-cell-deficient mice (40). Other microbially derived metabolites, including tryptophan metabolites and bile acid derivatives, are also altered in RA and may contribute to disease pathogenesis (41, 42). For example, elevated levels of certain tryptophan metabolites, such as indole derivatives, have been detected in the blood of individuals at risk of RA, and some microbially produced indoles can enhance Th17 responses and aggravate arthritis in mice (40). Additional fermentation products, such as succinate produced by Bacteroides, Prevotella, and Clostridium, may act as signaling molecules that activate dendritic cells and induce IL-1β and IL-6 production, thereby amplifying Th17 immunity. In CIA models, succinate accumulation enhances Th17-promoting pathways, whereas deletion of the host succinate receptor attenuates disease severity (43, 44). Taken together, altered microbial metabolites are integral to the gut–joint axis: loss of beneficial metabolites such as SCFAs weakens immunoregulatory functions, whereas accumulation of pro-inflammatory metabolites may promote the onset and progression of RA (33, 45).

## 3 Microbial community shifts and studies of specific strains

### 3.1 Overall features of the RA gut microbiota

Accumulating evidence indicates that the intestinal microbial profile of patients with RA differs substantially from that of healthy individuals (22, 46). In general, RA is associated with reduced microbial diversity and ecological imbalance, characterized by loss of beneficial SCFA-producing taxa and expansion of potentially pathogenic organisms (14, 47). Similar findings were confirmed by a systematic review of 92 observational studies, which showed that several rheumatic diseases, including RA, share the feature of reduced gut microbial α-diversity (48). At the taxonomic level, many studies have reported reduced abundance of Bacteroides together with a relative increase in Prevotella, a pattern considered a hallmark of early dysbiosis in RA (13). For example, Scher and colleagues first reported a marked expansion of Prevotella copri in the feces of patients with new-onset RA, and the abundance of this bacterium was linked to increased susceptibility to arthritis (49). Prevotella enrichment has also been observed during the preclinical phase, including in anti-CCP-positive individuals and subjects at high risk of RA (50). Cohort studies further suggest that genetically susceptible but clinically unaffected individuals already show microbiome alterations suggestive of RA, such as enrichment of P. copri and depletion of beneficial commensals including Bacteroides (13, 14).

It is important to note that host genetic background may interact with Prevotella enrichment. Alpízar-Rodríguez and colleagues reported that carriers of high-risk HLA-DRB1 susceptibility alleles had relatively lower levels of gut Prevotella, whereas individuals without these alleles showed higher Prevotella abundance, suggesting that Prevotella may, to some extent, compensate for genetic risk during disease initiation (51). In other words, individuals lacking strong genetic susceptibility may still experience an increased burden of Prevotella that contributes to RA development (52). This observation may help explain inter-cohort differences in reported Prevotella abundance and further highlights gene–microbiome interactions in RA pathogenesis.

### 3.2 Other taxa increased in RA

In addition to Prevotella, other Actinobacteria have also been reported to increase in the RA gut, particularly the genera Collinsella and Eggerthella. Metagenomic profiling by Chen et al. revealed enrichment of these taxa in patients with RA (53, 54). Collinsella has attracted particular attention because its abundance is associated with RA disease activity (55), and functional studies suggest that it may contribute to inflammation by increasing intestinal permeability and enhancing inflammatory signaling (56). Oral administration of Collinsella in CIA mice impaired gut barrier function, increased circulating pro-inflammatory cytokines, and aggravated arthritis (57), suggesting that its expansion may be pathogenic rather than merely secondary to inflammation.

Lactobacillus has also been reported to increase in certain RA cohorts (58). Although Lactobacillus is generally considered beneficial, its role in autoimmunity appears to be context dependent. Some studies reporting enrichment of particular Lactobacillus species in active RA suggest that these changes may reflect selective pressure imposed by the inflammatory environment (58). In contrast, some interventions, such as high-fiber dietary modulation, are associated with reduced Lactobacillus abundance together with symptom improvement (59). Thus, the contribution of Lactobacillus to RA has not been fully established and is likely to be strain specific, as discussed further in the probiotic section below.

### 3.3 Depleted beneficial taxa

In contrast to taxa enriched in RA, patients often exhibit reduced abundance of Bifidobacterium, Bacteroides, and several butyrate-producing taxa within the families Ruminococcaceae and Bacteroidaceae compared with healthy controls (14, 60). Bacteroides is a dominant commensal genus in the human gut and contributes to fermentation of dietary fiber and maintenance of mucosal tolerance (14, 61). These beneficial commensals are often depleted in early RA (13, 14). Studies indicate that untreated new-onset RA is associated with marked loss of Bacteroides, whereas effective control of disease activity is accompanied by partial recovery of Bacteroides abundance toward a healthier microbial profile (62). These findings suggest that active inflammation may be associated with depletion of Bacteroides, while therapeutic response may partly restore microbiota homeostasis.

Other important SCFA producers, such as Firmicutes including Faecalibacterium prausnitzii, are also commonly diminished in RA (63). Faecalibacterium is traditionally regarded as an anti-inflammatory commensal, and its depletion may reflect loss of immunoprotective metabolite production. Overall, recurrent disturbances in the RA intestinal microbiome are characterized by depletion of anti-inflammatory or SCFA-producing commensals, such as Bacteroides and Bifidobacterium, together with expansion of potentially pro-inflammatory taxa, including Prevotella and Collinsella (14, 47). However, taxonomic changes may vary across studies according to ethnicity, geography, diet, and other factors, and some genera show inconsistent patterns between cohorts. This variability underscores the need to define microbial signatures in diverse populations and across different disease stages (64, 65).

### 3.4 Functional studies of specific strains

To move beyond simple association toward causality, mechanistic studies have focused on strains closely linked to RA. Prevotella copri is the most extensively investigated example. In addition to its enrichment in new-onset RA, experimental evidence supports a pathogenic role: in SKG mice, a spontaneous autoimmune arthritis model, gut colonization with P. copri from patients with RA promotes dendritic-cell maturation and elicits strong Th17 responses, including enhanced IL-17 reactivity to self-antigens (66, 67). Human studies have also identified a 27-kDa P. copri protein (Pc-p27) and shown that many patients with RA produce anti-P. copri IgA and IgG antibodies (67, 68). These findings suggest that P. copri can provide antigenic stimuli capable of eliciting specific immune responses and may participate in molecular mimicry, as discussed below (67, 69).

Importantly, not all Prevotella species are deleterious. Prevotella histicola is a commensal bacterium naturally present in the human gut and has shown protective effects in RA models. Marietta et al. reported that administration of P. histicola suppressed inflammatory arthritis in humanized mice (70), possibly through the induction of regulatory immune programs (71). This contrast highlights the fact that functional properties may differ markedly even within the same genus and that microbiome-based therapies must be developed at the strain level rather than the genus level (72).

Another genus of interest is Parabacteroides, particularly Parabacteroides distasonis, a member of the order Bacteroidales. Depletion of Parabacteroides in patients with RA is notable, and its abundance is negatively correlated with disease activity (73). Sun et al. (2023) showed that oral administration of P. distasonis significantly alleviated arthritis in CIA mice (73, 74). Mechanistically, P. distasonis appeared to help restore microbial community balance, correct the Th17/Treg imbalance, and reduce pro-inflammatory cytokine production, thereby exerting anti-inflammatory effects (73, 75). These findings identify P. distasonis as a potential microbiome-based therapeutic target.

Several Lactobacillus and Bifidobacterium strains have also shown protective effects. Fan and colleagues reported that Bifidobacterium adolescentis reduced arthritis severity in CIA rats, although the benefit was time dependent and prophylactic administration before disease induction was most effective (76). Jhun et al. found that Lactobacillus sakei mitigated Th17 differentiation and promoted regulatory B-cell responses, thereby attenuating murine arthritis (68). Together, these studies suggest that selected probiotic strains possess immunomodulatory potential and may serve as adjunctive candidates in RA. However, strain-to-strain variability is substantial: although some Lactobacillus strains are beneficial, Lactobacillus expansion has also been reported in certain RA cohorts (77). Therefore, strain-level functional characterization is essential to distinguish beneficial from potentially harmful contributors and to guide personalized intervention.

## 4 Induction and maintenance of autoimmune mechanisms

### 4.1 Molecular mimicry and antigen-specific immunity

Gut-colonizing microbes may trigger autoimmunity through molecular mimicry, whereby microbial antigens share structural similarity with host self-antigens and thereby activate autoreactive T or B cells (69). Several lines of evidence support this mechanism in RA. For example, Maeda et al. showed that the commensal P. copri enhances Th17 responses against the self-joint antigen RPL23A (ribosomal protein L23a) in SKG mice (67). Human studies further indicate that T cells from patients with RA recognize P. copri-derived peptides and that B cells produce anti-P. copri antibodies, suggesting epitope similarity between P. copri antigens and joint-associated host constituents (67). Other studies have identified RA-relevant self-antigens targeted by homologous peptides from Prevotella and Parabacteroides, as well as sequences resembling FLNA (filamin A)-derived epitopes in Prevotella and Butyricimonas (78). Together, these findings suggest that intestinal microbes may provide disguised antigens that provoke T- and B-cell responses cross-reactive with joint tissues (78).

Metagenomic studies by Zhang Xiang et al. also found that a substantial proportion of microbial gene products in the RA gut share sequence homology with self-antigens, including cartilage collagen XI and peptides encoded by selected HLA-DR loci, and that genes potentially involved in molecular mimicry are enriched in RA-associated microbiomes (79). Oral and gut microbes may also contribute: protein fragments from Candida and Streptococcus can cross-react with immunodominant epitopes of type II collagen, a major RA autoantigen, and these microbial components exacerbate collagen-induced arthritis in mice (80). Thus, multiple microbial antigens may mimic joint constituents and initiate autoimmune cascades.

In addition to direct mimicry, microbial invasion or infection may promote host-cell stress and cell death, leading to release and processing of intracellular antigens that can be perceived as non-self and stimulate autoantibody production (81). Some oral pathogens express peptidylarginine deiminase (PAD) enzymes that catalyze protein citrullination, and PAD homologs have been identified across the human microbiome (82). Anti-citrullinated protein antibodies (ACPAs) may therefore target both host-derived and microbe-derived citrullinated proteins. Indeed, autoantibodies against citrullinated gut microbial proteins have been detected in patients with RA (83). These observations support the concept that gut microbes can provide noncanonical antigenic stimuli that breach immune tolerance and promote highly specific autoimmune responses in susceptible hosts.

### 4.2 Imbalance of Th17/Th1 and Treg responses

CD4+ T-cell dysregulation is a hallmark of RA and is characterized by overactivity of pro-inflammatory Th17 and Th1 cells together with insufficient regulatory T-cell function. The gut microbiota plays a critical role in shaping this imbalance. As discussed above, certain taxa, including segmented filamentous bacteria (SFB) and P. copri, can potently drive intestinal Th17 differentiation; these Th17 cells may subsequently migrate to synovial tissue, where they secrete IL-17 and related cytokines and thereby exacerbate joint inflammation (6, 26). Classic studies with SFB demonstrated that in K/BxN mice, another autoimmune arthritis model, arthritogenic Th17 responses arise only when the gut is colonized by SFB, whereas germ-free mice or mice lacking SFB show minimal arthritis (26).

Differentiation of these pro-inflammatory T cells often depends on cytokines produced by intestinal antigen-presenting cells, including IL-23, IL-1, and IL-6, thereby establishing the IL-23/IL-17 axis within gut–joint immunity. Experimental evidence indicates that IL-23 deficiency confers resistance to microbe-induced arthritis, underscoring the central role of IL-23-mediated Th17 expansion in microbiota-driven pathogenicity. For example, the microbial metabolite succinate can stimulate dendritic cells to secrete IL-1 and IL-6, thereby amplifying Th17 responses and worsening arthritis in mice (43). Blockade of succinate signaling or inhibition of IL-1 pathways mitigates inflammation, consistent with suppression of the Th17 axis (44).

Th1 cells, which produce IFN-γ, also contribute to RA pathogenesis, and certain gut microbes, including specific Clostridium species, may promote Th1-biased immune responses. Nonetheless, current evidence suggests that Th17 cells are the dominant microbiota-linked drivers of T-cell-mediated immune abnormalities in RA (17).

By contrast, Treg cells maintain peripheral immune tolerance and suppress excessive immune activation. Under healthy conditions, Treg homeostasis is supported by microbial metabolites, including SCFAs and vitamin-related compounds (37). In RA, however, inflammation and dysbiosis may reduce both the number and function of Treg cells. Wu and colleagues reported that a specialized Treg subset, Tfr cells, is significantly reduced in RA and is associated with microbial dysbiosis and altered metabolites (84). Reduced Tfr activity may fail to restrain Tfh cells and B cells, thereby permitting uncontrolled autoantibody production. Impaired peripheral induction of Treg cells may be further aggravated by depletion of key metabolites such as butyrate (85). In murine studies, butyrate supplementation promotes colonic Foxp3+ Treg expansion and IL-10 production, thereby providing protection during the early stages of arthritis (39, 86). Other reports suggest that some probiotics, including Bifidobacterium and Lactobacillus, can increase peripheral Treg proportions and inhibit arthritis development (77). Taken together, microbiota-driven shifts in the Th17/Treg balance toward Th17 predominance and reduced Treg activity may induce and sustain autoreactivity, thereby contributing to chronic RA.

A healthy gut ecosystem helps maintain regulatory T-cell (Treg) homeostasis through short-chain fatty acids (SCFAs), such as butyrate, and vitamin-related mechanisms that promote IL-10 production and Foxp3+ Treg stability. In RA-associated dysbiosis, reduced levels of SCFA-producing microorganisms and impaired Treg support weaken IL-10-mediated suppression and permit expansion of follicular helper T cells and B-cell differentiation, thereby promoting production of autoantibodies such as rheumatoid factor (RF) and anti-citrullinated protein antibodies (ACPAs) and contributing to systemic autoimmunity. Peripheral Treg responses may be partially restored by probiotics such as Bifidobacterium and Lactobacillus, helping to re-establish effector–regulatory immune balance and slow inflammation. This gut–Th17/Treg–joint axis therefore represents a fundamental metabolic–immune pathway in RA and an attractive target for preventive and therapeutic intervention.

### 4.3 Tfh cells and B-cell responses

Autoantibody production, including anti-citrullinated protein antibodies (ACPAs) and rheumatoid factor (RF), is a hallmark of RA and depends on B-cell activation and differentiation within lymphoid follicles. Growing evidence indicates that gut microbes can shape these humoral immune processes (87, 88). Teng and colleagues demonstrated that the gut microbiota can induce Tfh-cell differentiation and promote migration to Peyer’s patches, thereby enhancing germinal-center responses and autoantibody production (89, 90). In the K/BxN model, depletion of the gut microbiota had little effect on Th17 cells but significantly reduced Tfh cells, which was accompanied by marked improvement in arthritis (91). These findings suggest that in some autoimmune settings, microbiota-mediated disease may operate primarily through the Tfh–B-cell axis rather than through Th17 cells alone.

Human studies also support a mucosal origin of autoantibody responses in RA. Using ileal biopsies, Derksen and colleagues detected locally secreted IgA and IgG autoantibodies in patients with RA, suggesting that the gut mucosa may serve as a site of autoantibody generation (92). Dysbiosis may promote excessive activation of Tfh and B cells within gut-associated lymphoid tissue. Even before clinical symptoms arise, serum IgA against gut microbes has been detected in some individuals at high risk of RA, indicating early mucosal immune dysregulation (88). Notably, Chriswell et al. cloned IgA and IgG antibodies from individuals at high risk of RA and found that these antibodies targeted a strain of Subdoligranulum; importantly, the antibodies induced arthritis in a mouse model, directly linking gut bacteria to antigen-specific, arthritogenic B-cell responses (88).

Microbial products can also directly modulate B-cell function. SCFAs are able to stabilize anti-inflammatory IL-10+ regulatory B cells and suppress migratory B-cell programs, thereby limiting early autoimmune escalation (39, 93). Overall, gut microbes may trigger and sustain RA autoimmunity not only through cross-reactive antigen presentation and activation of autoreactive B and T cells, but also by reshaping helper T-cell support for B cells within the mucosal immune environment.

## 5 Advances in microecology-based interventions

### 5.1 Probiotic therapy

Restoration of gut ecological balance through beneficial microorganisms is one of the principal strategies in microbiology-based intervention for RA. Several small randomized controlled trials (RCTs) have evaluated probiotic supplementation in RA. Zamani et al. (2019), in a double-blind RCT, reported that eight weeks of a multi-strain probiotic containing Lactobacillus acidophilus, L. casei, and Bifidobacterium bifidum significantly reduced Disease Activity Score in 28 joints (DAS28) and C-reactive protein (CRP) compared with placebo (94, 95). Two other independent RCTs, including those by Vaghef-Mehrabany et al. and Alipour et al., likewise found that eight weeks of L. casei supplementation improved clinical measures, including tender and swollen joint counts and inflammatory markers, relative to placebo (96).

However, a 2018 meta-analysis of four trials reached a more cautious conclusion: overall, probiotic interventions did not produce statistically significant improvements over placebo for major clinical outcomes (97). The authors emphasized that interpretation was limited by small sample sizes and substantial heterogeneity across studies. These findings suggest that the clinical reproducibility of probiotic benefits in RA remains to be established (97).

Animal studies provide stronger support for beneficial probiotic effects in arthritis models than do the mixed findings from human trials (77). Bifidobacterium, including B. adolescentis and related strains, has been shown to increase peripheral Treg proportions, restore immune balance, and reduce arthritis incidence in preclinical RA settings, particularly during the pre-RA phase of susceptible models (77). Lactobacillus helveticus and P. histicola have likewise been reported to suppress arthritis by modulating T-helper-cell differentiation, inhibiting Th17/Tfh responses, promoting Treg activity, and regulating both local and systemic B-cell responses (98).

Notably, some studies suggest that probiotic interventions are most effective during early or preventive phases; once arthritis becomes chronic, probiotics alone may be insufficient to reverse established immunopathology (99). This observation implies that probiotics may be more useful as preventive or adjunctive therapy than as stand-alone treatment for late-stage RA. Overall, probiotic effects in RA appear to be both strain specific and time dependent: some strains, such as L. casei and B. longum, may improve clinical and inflammatory indices when administered at appropriate stages, but their efficacy is strongly influenced by host factors, strain combinations, dosage, and treatment duration (100).

### 5.2 Prebiotics and postbiotics

In addition to live microbial supplementation, dietary modulation of the microbiota (prebiotics) and the use of microbial products (postbiotics) represent promising complementary approaches. Dietary fiber and oligosaccharides are among the most extensively studied prebiotics. Inulin is a fermentable prebiotic that promotes the growth of beneficial bacteria in the gut. In animal models, pre-arthritis administration of inulin reduces joint swelling and slows disease progression (101), likely by enriching beneficial taxa such as Lactobacillus and Bifidobacterium while creating an unfavorable environment for pro-inflammatory organisms. Resistant starch, another poorly digestible substrate, is fermented in the colon and generates substantial amounts of SCFAs. High-resistant-starch diets can prevent arthritis onset in CIA mice (102). Bai et al. further reported that resistant starch enhanced propionate production and improved microbial composition, thereby attenuating arthritis (103).

In a dietary intervention study involving 36 patients with RA, only four weeks of fiber enrichment led to favorable biological changes, including reduced serum zonulin, increased Treg abundance, lower Th1/Th17 ratios, and reduced levels of biomarkers associated with bone erosion (28, 104). These findings support the concept that high-fiber intake may exert multi-level benefits in RA by remodeling the microbiota and increasing metabolite production. Other functional food components with prebiotic-like properties have also been investigated. Oral lactoferrin administration markedly inhibited disease progression in SKG mice (105). Spirulina supplementation was reported to improve dysbiosis in adjuvant-induced arthritis (AIA) rats, lower TNF-α and IL-6 levels, and consequently reduce arthritis severity (106). Together, these data suggest that selected dietary components may exert indirect anti-arthritic effects through regulation of microbial ecology.

Postbiotics are bioactive microbial metabolites and/or inactivated microbial preparations that bypass the variability associated with live bacterial colonization while retaining beneficial immunologic properties. Several postbiotics have shown efficacy in RA models. For example, heat-killed Propionibacterium administered to CIA mice inhibited osteoclast differentiation and reduced bone destruction, thereby exerting joint-protective effects (107). Similarly, administration of inactivated Lactiplantibacillus plantarum and its culture supernatant reduced rheumatoid factor and ACPA titers, inflammatory cytokine levels, and tissue damage in arthritis models (108). These studies suggest that non-pathogenic microbial components and metabolites can modulate host immunity and attenuate RA-associated inflammation even in the absence of live bacteria. SCFAs are a representative class of postbiotics and are generally regarded as particularly promising for clinical translation.

### 5.3 Fecal microbiota transplantation

Fecal microbiota transplantation (FMT) involves transfer of fecal microbiota from healthy donors to recipients in order to restore a healthier microbial ecosystem and is an established treatment for recurrent Clostridioides difficile infection. In RA, however, FMT remains investigational, although several case reports and pilot studies have been published. A particularly encouraging early example was reported by Zeng et al., in which repeated administration of encapsulated donor microbiota was associated with sustained symptomatic and laboratory improvement for more than six months (109, 110). Subsequent preliminary case series from China also suggested potential benefit in refractory RA, but large controlled studies are still lacking. Ongoing registered trials primarily evaluate feasibility, safety, and microbiome-related outcomes; one example is an RA FMT study using freeze-dried capsules (100).

Mechanistic animal studies provide additional support for the relevance of microbiota transfer. Transplantation of microbiota from patients with RA or from individuals at high risk of RA into germ-free mice impairs gut barrier integrity, promotes Th17 bias, and increases susceptibility to arthritis (20, 22). By contrast, transplantation of microbiota from healthy donors may be protective [133]. In one study, transfer of microbiota from pre-RA individuals into CIA mice reduced tight-junction protein expression, increased Th17 cells and other inflammatory mediators, and worsened arthritis (20, 22). These findings suggest that autoimmune susceptibility can be transmissible through a pathogenic microbiota. In principle, transplantation of a healthy microbiota into patients with RA could help restore mucosal immune homeostasis and reduce systemic inflammation, although several practical challenges remain (111).

Safety remains a major concern because many patients with RA receive immunosuppressive therapy, which may increase susceptibility to infection and other adverse events; therefore, rigorous donor screening and post-procedure surveillance are essential (111). Second, the long-term durability of FMT effects is uncertain, and it remains unclear whether transplanted microbial communities can achieve stable engraftment and sustained functional benefit (111). Third, psychological barriers, together with the need for repeated monitoring and follow-up, may limit acceptability and adherence (111). Finally, studies in related diseases such as psoriatic arthritis indicate that FMT is not uniformly effective and may depend on disease subtype and donor–recipient matching (94, 112). Overall, FMT for RA remains in its infancy: although it offers a compelling concept of ecological reconstruction, the heterogeneity of RA suggests that a single course of FMT is unlikely to be universally sufficient and may need to be combined with other therapeutic strategies.

## 6 Current challenges and future directions

Although knowledge of the role of gut microbes in RA is accumulating rapidly, substantial gaps remain and must be addressed systematically (113).

Causality and individual variability remain major challenges. Most available studies are cross-sectional, making it difficult to determine whether microbiota shifts are causes, consequences, or both. Because host genetics, diet, geography, and lifestyle exert profound effects on the microbiome, inter-individual variation may exceed disease-related effects in some cohorts (64). As a result, microbiome signatures are not always consistent across populations: a genus may be enriched in one cohort but not in another. Future work therefore requires well-designed prospective longitudinal studies that follow high-risk individuals over time in order to identify microbial changes that precede disease onset (114). In addition, population heterogeneity, including age, diet, and medication exposure, should be carefully controlled or statistically adjusted to reduce confounding (65). Such rigor is essential for moving beyond correlation toward causal inference and for identifying truly pathogenic or protective taxa. Future models should also integrate diet, medication, host genetics, and disease stage, and machine-learning approaches may help improve patient stratification and biomarker discovery when supported by robust external validation.

A deeper mechanistic framework is also needed. Mechanisms cannot be inferred from association alone. Although several conceptual models have been proposed, including molecular mimicry, barrier disruption, and metabolite-mediated regulation, many molecular pathways remain incompletely understood. Future studies should define microbe–immune-cell interactions at both molecular and cellular levels. Key questions include which microbial antigens drive Th17 or Treg differentiation, which signaling pathways underlie these effects, and which pattern-recognition receptors are engaged by specific taxa. Germ-free recolonization, microbiota transplantation, and mono-colonization models remain valuable tools for addressing these questions, particularly when combined with contemporary immunology and molecular biology approaches (17, 26). Multi-omics strategies, including metagenomics, metatranscriptomics, metabolomics, and immune profiling, should also be leveraged to identify critical nodes within microbe–metabolite–host immune networks. For example, metabolomics can identify candidate metabolites involved in RA pathogenesis, which can then be traced back to their microbial origins to reveal new pathogenic factors (41, 111).

Improved resolution of microbiome characterization is another priority. Many RA microbiome studies still rely on 16S rRNA sequencing, which typically resolves taxa only to the genus or family level (111). However, microbial functions are often species- or even strain-specific: different strains within the same genus may be either pathogenic or beneficial (72). For example, P. copri is often pro-inflammatory, whereas P. histicola appears protective (70), and immunomodulatory activity varies markedly among Lactobacillus strains (111). Consequently, genus-level shifts alone may be insufficient to identify the true biological drivers. Future studies should therefore employ deep shotgun metagenomic sequencing together with cultivation-based approaches to identify key organisms at the species and strain levels. Strain-specific determinants, such as unique gene clusters or metabolic pathways, may then inform development of targeted probiotics or strategies to block specific pathobionts (72).

Finally, non-bacterial members of the gut microbiome deserve greater attention. Fungi and viruses, particularly bacteriophages, may also influence RA pathogenesis, but they remain understudied (115). Available evidence suggests that the gut phageome differs between individuals at high risk of RA and healthy controls, implying that phages may indirectly modulate inflammation by shaping their bacterial hosts (111). Future studies should therefore adopt a more holistic microbiome perspective encompassing bacteria, fungi, and viruses. Emerging technologies such as single-cell transcriptomics, spatial profiling, and integrated host–microbe multi-omics may further clarify cell-specific mechanisms within the gut–joint axis and provide a more complete view of disease biology.

## 7 Concluding remarks

Gut microbiota-driven autoimmunity in RA represents a complex but rapidly evolving field. In recent years, research has progressed from observational association toward early mechanistic insight and exploratory intervention. These advances have deepened our understanding of RA pathogenesis and opened new possibilities for prevention and treatment (116). Continued progress will require higher-resolution microbiome profiling, longitudinal and causal study designs, and integrative multi-omics supported by functional experimentation. As the field advances, gut microecology may become an increasingly important component of RA management and may help improve disease control and therapeutic response.

**Figure 1:**
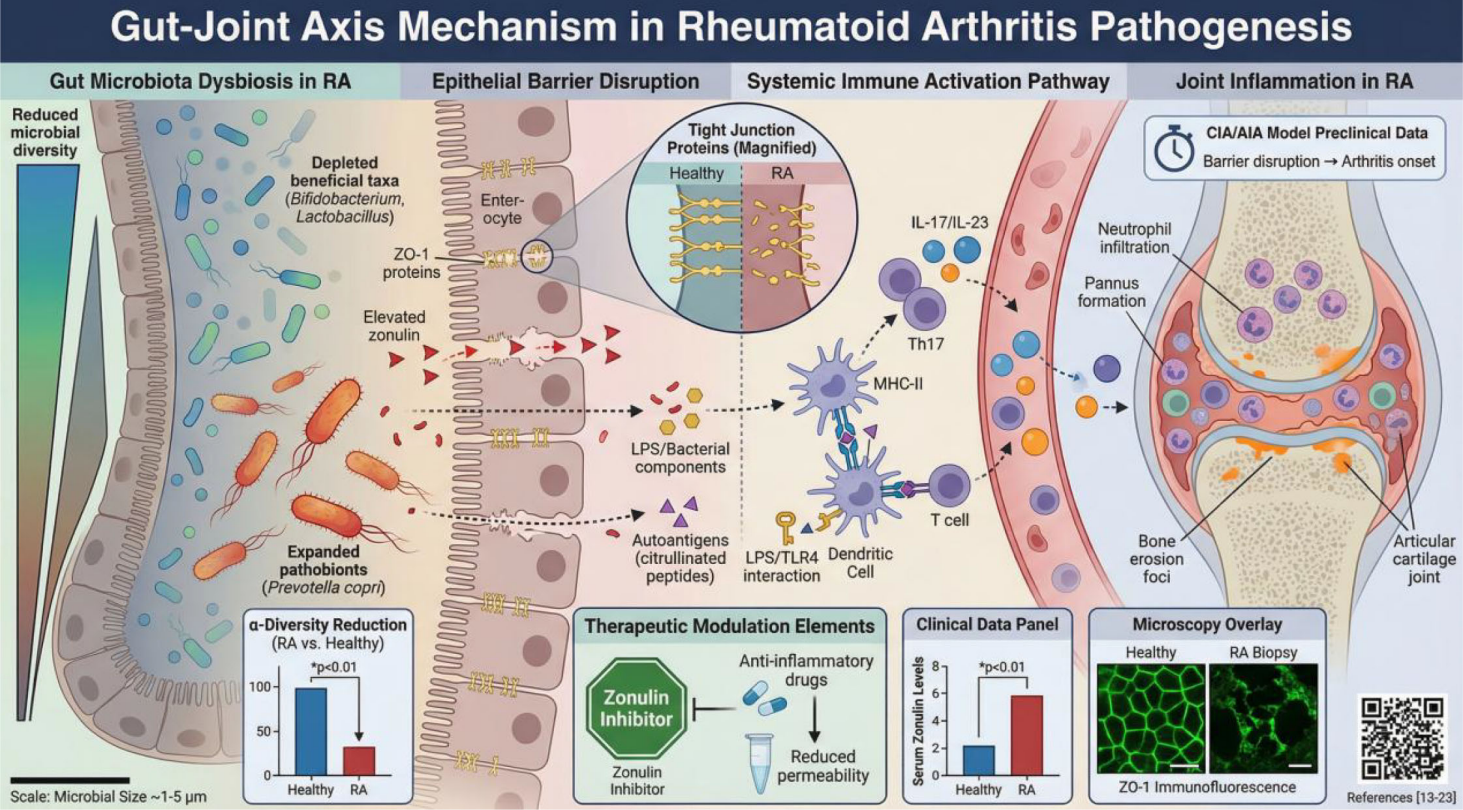
Illustrates the gut–joint axis in rheumatoid arthritis (RA) driven by gut microbiota dysbiosis. Alterations in gut microbial community structure, including reduced diversity, loss of beneficial taxa such as Bifidobacterium and Lactobacillus, and expansion of putative pathobionts, promote intestinal microecological imbalance. Dysbiosis impairs epithelial barrier integrity by disrupting tight-junction proteins such as ZO-1, increasing zonulin expression, and enhancing intestinal permeability. As a consequence, microbial products and luminal antigens can cross the intestinal barrier and enter the circulation, where they trigger innate and adaptive immune responses. Antigen presentation and innate immune sensing activate dendritic cells and T cells, enhance Th17 polarization, and increase pro-inflammatory cytokine signaling, including IL-17 and IL-23. These events amplify autoantigen reactivity, recruit inflammatory cells, and ultimately contribute to synovial inflammation, pannus formation, bone erosion, and cartilage destruction. The schematic also highlights translational evidence from CIA/AIA models and clinical studies of zonulin and ZO-1, as well as potential therapeutic strategies targeting barrier dysfunction and inflammatory pathways.

**Figure 2:**
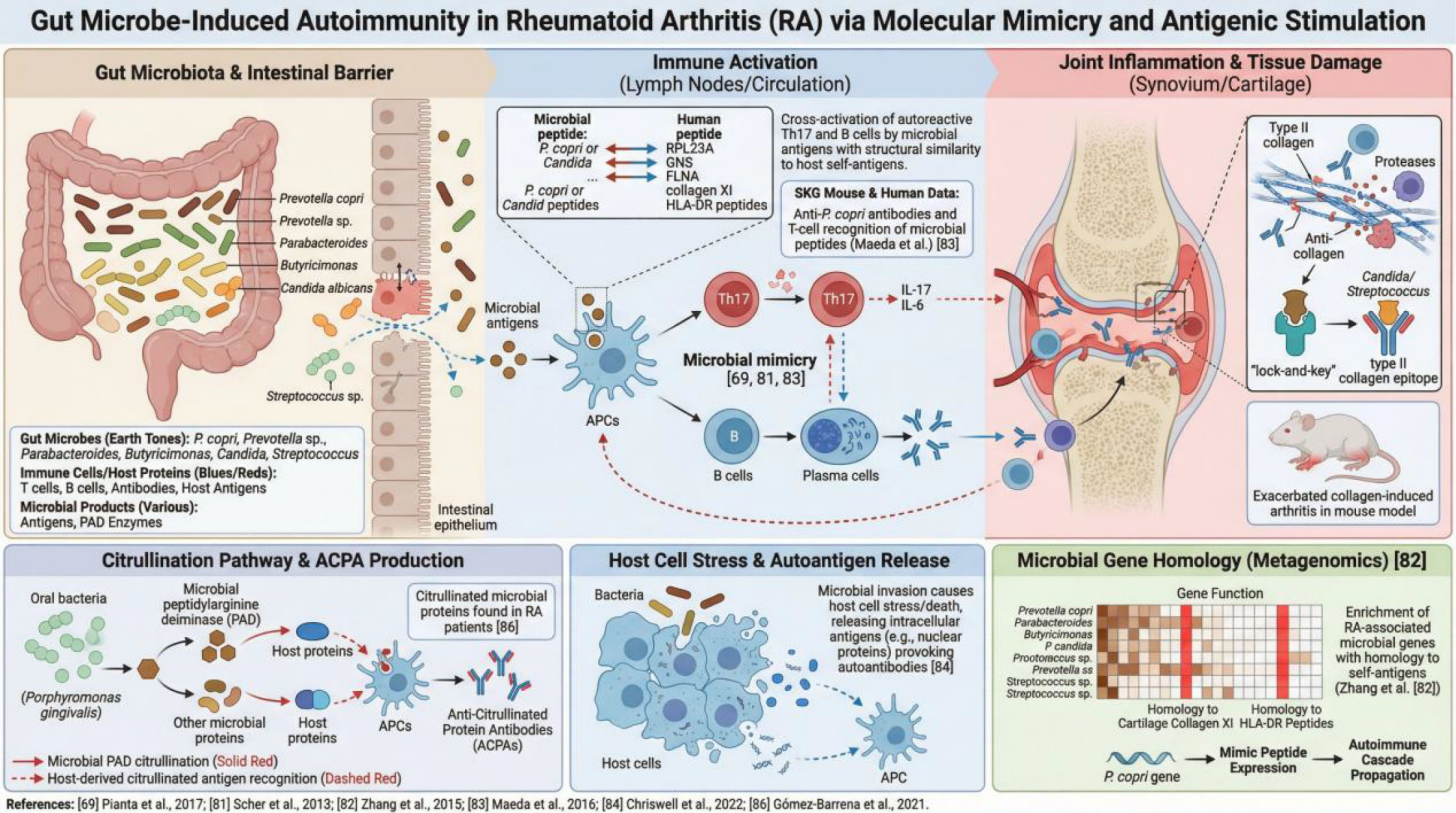
Summarizes how gut microbiota may drive autoimmunity in rheumatoid arthritis (RA) through molecular mimicry, antigenic stimulation, and immune dysregulation. Microbes and their antigens in the intestine may contribute to RA pathogenesis by disrupting mucosal barrier integrity and altering host immune homeostasis. Dysbiosis may facilitate translocation of bacterial constituents and peptides from taxa such as Prevotella copri, Parabacteroides, Butyricimonas, Candida albicans, and Streptococcus spp., which are taken up by antigen-presenting cells and promote activation of Th17 cells and autoreactive B cells. Cross-reactive immune responses may then arise through molecular mimicry and sequence homology between microbial products and host proteins, including type II collagen, thereby favoring autoantibody production and inflammatory amplification. Citrullinated microbial and host proteins generated in dysbiotic conditions may further stimulate anti-citrullinated protein antibody (ACPA) responses. At the same time, microbial imbalance can induce host-cell stress and release of autoantigens, further aggravating the loss of immune tolerance. Increased IL-17, IL-6, and other pro-inflammatory mediators within the joint microenvironment then drive synovial inflammation, protease release, and cartilage destruction. Reduced production of immunoregulatory microbial metabolites and loss of barrier-protective functions further amplify immune disequilibrium. Overall, this gut–immune–joint axis represents a key mechanistic model for RA initiation and progression and highlights opportunities for microbiota-targeted intervention.

**Figure 3:**
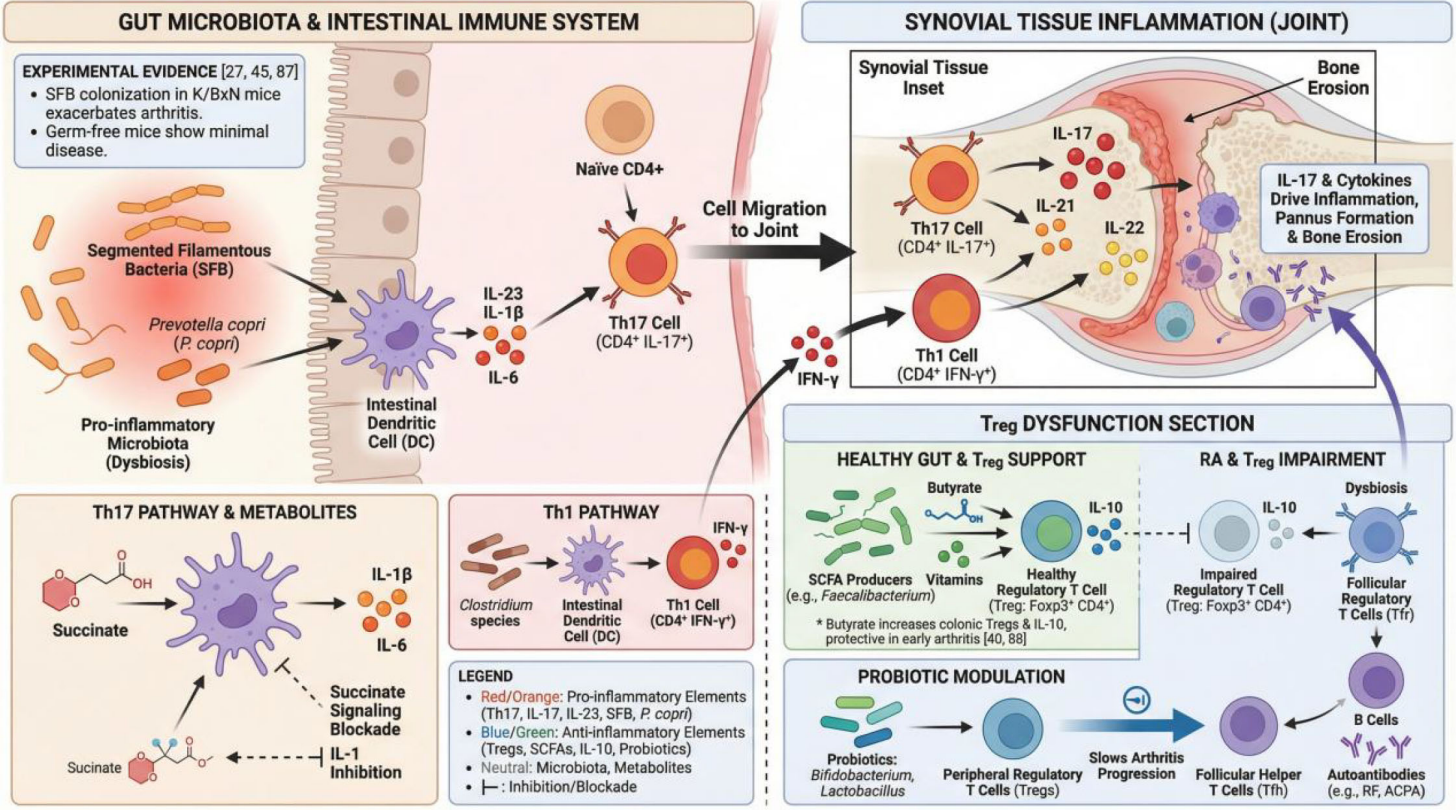
Illustrates how gut microbiota dysbiosis and intestinal immune imbalance may contribute to rheumatoid arthritis (RA). The intestinal microbiota and their metabolites play central roles in regulating systemic immunity in RA. Expansion of pro-inflammatory taxa, such as segmented filamentous bacteria and Prevotella copri, activates intestinal dendritic cells and increases production of IL-23, IL-1β, and IL-6, thereby promoting polarization of naïve CD4+ T cells toward Th17 cells. These activated Th17 cells may then migrate to the joints and secrete IL-17, IL-21, and IL-22, further amplifying local cytokine cascades, sustaining synovial inflammation, and promoting pannus formation and bone erosion. In parallel, metabolite-mediated signaling, including pro-inflammatory effects of succinate, may further enhance Th17 activation, whereas inhibition of these pathways can attenuate upstream inflammatory priming.

## References

[1] Smolen JS Aletaha D McInnes IB . Rheumatoid arthritis. Lancet. (2016) 388(10055):2023–38. doi: 10.1016/S0140-6736(16)30173-8. PMID: 27156434

[2] Yang W Yang W Chen J Chen J Wang X Wang X . Rheumatoid arthritis: emerging insights into molecular mechanisms and targeted immunotherapy. Clin Exp Rheumatol. (2026) 44(1):147–55. doi: 10.55563/clinexprheumatol/dgbqaa. PMID: 40892670

[3] MacGregor AJ Snieder H Rigby AS Koskenvuo M Kaprio J Aho K . Characterizing the quantitative genetic contribution to rheumatoid arthritis using data from twins. Arthritis Rheum. (2000) 43(1):30–7. doi: 10.1002/1529-0131(200001)43:1<30::AID-ANR5>3.0.CO;2-B. PMID: 10643697

[4] Choi HG Kim SY Kwon BC Kang HS Lim H Kim J-H . Comparison of the coincidence of osteoporosis, fracture, arthritis histories, and DEXA T-score between monozygotic and dizygotic twins: a cross-sectional study using KoGES HTS data. Nutrients. (2022) 14(18):3836. doi: 10.3390/nu14183836. PMID: 36145209 PMC9506177

[5] Zouali M . B lymphocytes, the gastrointestinal tract and autoimmunity. Autoimmun Rev. (2021) 20(4):102777. doi: 10.1016/j.autrev.2021.102777. PMID: 33609796

[6] Arifuzzaman M Collins N Guo CJ . Nutritional regulation of microbiota-derived metabolites: implications for immunity and inflammation. Immunity. (2024) 57(1):14–27. doi: 10.1016/j.immuni.2023.12.009. PMID: 38198849 PMC10795735

[7] Scher JU Sczesnak A Longman RS Segata N Ubeda C Bielski C . Expansion of intestinal *Prevotella copri* correlates with enhanced susceptibility to arthritis. Elife. (2013) 2:e01202. doi: 10.7554/eLife.01202. PMID: 24192039 PMC3816614

[8] Alpizar-Rodriguez D Lesker TR Gronow A Gilbert B Raemy E Lamacchia C . *Prevotella copri* in individuals at risk for rheumatoid arthritis. Ann Rheum Dis. (2019) 78(5):590–3. doi: 10.1136/annrheumdis-2018-214514. PMID: 30760471

[9] Qi XY Liu MX Jiang XJ Gao T Xu GQ Zhang HY . Gut microbiota in rheumatoid arthritis: Mechanistic insights, clinical biomarkers, and translational perspectives. Autoimmun Rev. (2025) 24(12):103912. doi: 10.1016/j.autrev.2025.103912. PMID: 40825448

[10] Yu D Du J Pu X Zheng L Chen S Wang N . The gut microbiome and metabolites are altered and interrelated in patients with rheumatoid arthritis. Front Cell Infect Microbiol. (2021) 11:763507. doi: 10.3389/fcimb.2021.763507. PMID: 35145919 PMC8821809

[11] Sun H Guo Y Wang H Yin A Hu J Yuan T . Gut commensal *Parabacteroides distasonis* alleviates inflammatory arthritis. Gut. (2023) 72(9):1664–77. doi: 10.1136/gutjnl-2022-327756. PMID: 36604114

[12] Qiao J Zhang SX Chang MJ Cheng T Zhang JQ Zhao R . Specific enterotype of gut microbiota predicts clinical effect of methotrexate in patients with rheumatoid arthritis. Rheumatology (Oxford). (2023) 62(3):1087–96. doi: 10.1093/rheumatology/keac458. PMID: 35946529

[13] Ruiz-Limon P Mena-Vázquez N Moreno-Indias I Manrique-Arija S Lisbona-Montanez JM Cano-Garcia L . *Collinsella* is associated with cumulative inflammatory burden in an established rheumatoid arthritis cohort. Biomed Pharmacother. (2022) 153:113518. doi: 10.1016/j.biopha.2022.113518. PMID: 36076603

[14] Chen J Wright K Davis JM Jeraldo P Marietta EV Murray J . An expansion of rare lineage intestinal microbes characterizes rheumatoid arthritis. Genome Med. (2016) 8(1):43. doi: 10.1186/s13073-016-0299-7. PMID: 27102666 PMC4840970

[15] Wu R Wang D Cheng L Su R Li B Fan C . Impaired immune tolerance mediated by reduced Tfr cells in rheumatoid arthritis linked to gut microbiota dysbiosis and altered metabolites. Arthritis Res Ther. (2024) 26(1):21. doi: 10.1186/s13075-023-03260-y. PMID: 38218985 PMC10787489

[16] Rogier R Evans-Marin H Manasson J van der Kraan PM Walgreen B Helsen MM . Alteration of the intestinal microbiome characterizes preclinical inflammatory arthritis in mice and its modulation attenuates established arthritis. Sci Rep. (2017) 7(1):15613. doi: 10.1038/s41598-017-15802-x. PMID: 29142301 PMC5688157

[17] Audo R Sanchez P Rivière B Mielle J Tan J Lukas C . Rheumatoid arthritis is associated with increased gut permeability and bacterial translocation which are reversed by inflammation control. Rheumatology (Oxford). (2023) 62(3):1264–71. doi: 10.1093/rheumatology/keac454. PMID: 35946514

[18] Matei DE Menon M Alber DG Smith AM Nedjat-Shokouhi B Fasano A . Intestinal barrier dysfunction plays an integral role in arthritis pathology and can be targeted to ameliorate disease. Med. (2021) 2(7):864–883.e9. doi: 10.1016/j.medj.2021.04.013. PMID: 34296202 PMC8280953

[19] Tajik N Frech M Schulz O Schälter F Lucas S Azizov V . Targeting zonulin and intestinal epithelial barrier function to prevent onset of arthritis. Nat Commun. (2020) 11(1):1995. doi: 10.1038/s41467-020-15831-7. PMID: 32332732 PMC7181728

[20] Marietta EV Murray JA Luckey DH Jeraldo PR Lamba A Patel R . Suppression of inflammatory arthritis by human gut-derived *Prevotella histicola* in humanized mice. Arthritis Rheumatol. (2016) 68(12):2878–88. doi: 10.1002/art.39785. PMID: 27337150 PMC5125894

[21] Hecquet S Totoson P Martin H Algros MP Saas P Pais-de-Barros JP . Increased gut permeability and intestinal inflammation precede arthritis onset in the adjuvant-induced model of arthritis. Arthritis Res Ther. (2023) 25(1):95. doi: 10.1186/s13075-023-03069-9. PMID: 37280714 PMC10242991

[22] Luo Y Tong Y Wu L Niu H Li Y Su LC . Alteration of gut microbiota in individuals at high-risk for rheumatoid arthritis associated with disturbed metabolome and the initiation of arthritis through the triggering of mucosal immunity imbalance. Arthritis Rheumatol. (2023) 75(10):1736–1748. doi: 10.1002/art.42616. PMID: 37219936

[23] Audo R Sanchez P Rivière B Mielle J Tan J Lukas C . Rheumatoid arthritis is associated with increased gut permeability and bacterial translocation which are reversed by inflammation control. Rheumatology (Oxford). (2023) 62(3):1264–71. doi: 10.1093/rheumatology/keac454. PMID: 35946514

[24] Heidt C Kämmerer U Fobker M Rüffer A Marquardt T Reuss-Borst M . Assessment of intestinal permeability and inflammation biomarkers in patients with rheumatoid arthritis. Nutrients. (2023) 15(10):2386. doi: 10.3390/nu15102386. PMID: 37242269 PMC10221762

[25] Wen J Lyu P Stolzer I . Epithelial HIF2α expression induces intestinal barrier dysfunction and exacerbation of arthritis. Ann Rheum Dis. (2022) 81(8):1119–30. doi: 10.1136/annrheumdis-2021-222035. PMID: 35710307

[26] Lv J Hao P Hao P Zhou Y Liu T Liu T . Role of the intestinal flora-immunity axis in the pathogenesis of rheumatoid arthritis-mechanisms regulating short-chain fatty acids and Th17/Treg homeostasis. Mol Biol Rep. (2025) 52(1):617. doi: 10.1007/s11033-025-10714-w. PMID: 40544212

[27] Zhu J . T helper cell differentiation, heterogeneity, and plasticity. Cold Spring Harb Perspect Biol. (2018) 10(10):a030338. doi: 10.1101/cshperspect.a030338. PMID: 28847903 PMC6169815

[28] Wang J Shan Y Jiang Z Feng J Li C Ma L . High frequencies of activated B cells and T follicular helper cells are correlated with disease activity in patients with new-onset rheumatoid arthritis. Clin Exp Immunol. (2013) 174(2):212–220. doi: 10.1111/cei.12162. PMID: 23786438 PMC3828824

[29] Liu X Chen X Zhong B . Transcription factor achaete-scute homologue 2 initiates follicular T-helper-cell development. Nature. (2014) 507(7493):513–518. doi: 10.1038/nature12910. PMID: 24463518 PMC4012617

[30] McCarron MJ Marie JC . TGF-β prevents T follicular helper cell accumulation and B cell autoreactivity. J Clin Invest. (2014) 124(10):4375–86. doi: 10.1172/JCI76179. PMID: 25157822 PMC4191003

[31] Kim YU Lim H Jung HE Wetsel RA Chung Y . Regulation of autoimmune germinal center reactions in lupus-prone BXD2 mice by follicular helper T cells. PLoS One. (2015) 10(3):e0120294. doi: 10.1371/journal.pone.0120294. PMID: 25768299 PMC4358919

[32] Chung Y Tanaka S Chu F . Follicular regulatory T cells expressing Foxp3 and Bcl-6 suppress germinal center reactions. Nat Med. (2011) 17(8):983–988. doi: 10.1038/nm.2426. PMID: 21785430 PMC3151340

[33] Chung Y Tanaka S Chu F Nurieva RI Martinez GJ Rawal S . Foxp3+ follicular regulatory T cells control the germinal center response. Nat Med. (2011) 17(8):975–982. doi: 10.1038/nm.2425. PMID: 21785433 PMC3182542

[34] Wu H Chen Y Liu H Xu LL Teuscher P Wang S . Follicular regulatory T cells repress cytokine production by follicular helper T cells and optimize IgG responses in mice. Eur J Immunol. (2016) 46(5):1152–1161. doi: 10.1002/eji.201546094. PMID: 26887860 PMC4896226

[35] Abebaw D Akelew Y Adugna A Tegegne BA Teffera ZH Belayneh M . Immunomodulatory properties of the gut microbiome: diagnostic and therapeutic potential for rheumatoid arthritis. Clin Exp Med. (2025) 25(1):226. doi: 10.1007/s10238-025-01777-x. PMID: 40591032 PMC12214023

[36] Ritvo PG Churlaud G Quiniou V Ritvo PG Churlaud G Quiniou V . Tfr cells lack IL-2Rα but express decoy IL-1R2 and IL-1Ra and suppress the IL-1-dependent activation of Tfh cells. Sci Immunol. (2017) 2(15):eaan0368. doi: 10.1126/sciimmunol.aan0368. PMID: 28887367

[37] Round JL Mazmanian SK . Inducible Foxp3+ regulatory T-cell development by a commensal bacterium of the intestinal microbiota. Proc Natl Acad Sci U S A. (2010) 107(27):12204–12209. doi: 10.1073/pnas.0909122107. PMID: 20566854 PMC2901479

[38] Fan Z Yang B Ross RP Stanton C Shi G Zhao J . Protective effects of *Bifidobacterium adolescentis* on collagen-induced arthritis in rats depend on timing of administration. Food Funct. (2020) 11(5):4499–4511. doi: 10.1039/d0fo00077a. PMID: 32383727

[39] Liu X Zeng B Zhang J Li W Mou F Wang H . Role of the gut microbiome in modulating arthritis progression in mice. Sci Rep. (2016) 6(1):30594. doi: 10.1038/srep30594. PMID: 27481047 PMC4969881

[40] Wu HJ Ivanov II Darce J Hattori K Shima T Umesaki Y . Gut-residing segmented filamentous bacteria drive autoimmune arthritis via T helper 17 cells. Immunity. (2010) 32(6):815–827. doi: 10.1016/j.immuni.2010.06.001. PMID: 20620945 PMC2904693

[41] Teng F Klinger CN Felix KM Bradley CP Wu E Tran NL . Gut microbiota drive autoimmune arthritis by promoting differentiation and migration of Peyer's patch T follicular helper cells. Immunity. (2016) 44(4):875–888. doi: 10.1016/j.immuni.2016.03.013. PMID: 27096318 PMC5296410

[42] Block KE Zheng Z Dent AL Kee BL . Gut microbiota regulates K/BxN autoimmune arthritis through follicular helper T but not Th17 cells. J Immunol. (2016) 196(4):1550–1557. doi: 10.4049/jimmunol.1501904. PMID: 26783341 PMC4744513

[43] Maeda Y Kurakawa T Umemoto E Motooka D Ito Y Gotoh K . Dysbiosis contributes to arthritis development via activation of autoreactive T cells in the intestine. Arthritis Rheumatol. (2016) 68(11):2646–2661. doi: 10.1002/art.39783. PMID: 27333153

[44] Crost EH Coletto E Bell A . *Ruminococcus gnavus*: friend or foe for human health. FEMS Microbiol Rev. (2023) 47(2):fuad014. doi: 10.1093/femsre/fuad014. PMID: 37015876 PMC10112845

[45] Fan Z Ross RP Stanton C Hou B Zhao J Zhang H . *Lactobacillus casei* CCFM1074 alleviates collagen-induced arthritis in rats via balancing Treg/Th17 and modulating the metabolites and gut microbiota. Front Immunol. (2021) 12:680073. doi: 10.3389/fimmu.2021.680073. PMID: 34079556 PMC8165437

[46] Sato K Takahashi N Kato T . Aggravation of collagen-induced arthritis by orally administered *Porphyromonas gingivalis* through modulation of the gut microbiota and gut immune system. Sci Rep. (2017) 7(1):6955. doi: 10.1038/s41598-017-07196-7. PMID: 28761156 PMC5537233

[47] Tezuka H Abe Y Asano J Sato T Liu J Iwata M . Prominent role for plasmacytoid dendritic cells in mucosal T cell-independent IgA induction. Immunity. (2011) 34(2):247–257. doi: 10.1016/j.immuni.2011.02.002. PMID: 21333555

[48] Koh JH Lee EH Cha KH Pan CH Kim D . Factors associated with the composition of the gut microbiome in patients with established rheumatoid arthritis and its value for predicting treatment responses. Arthritis Res Ther. (2023) 25(1):32. doi: 10.1186/s13075-023-03013-x. PMID: 36864473 PMC9979421

[49] Luo Y Tong Y Wu L Niu H Li Y Su LC . Alteration of Gut Microbiota in Individuals at High-Risk for Rheumatoid Arthritis Associated With Disturbed Metabolome and the Initiation of Arthritis Through the Triggering of Mucosal Immunity Imbalance. Arthritis Rheumatol. (2023) 75(10):1736–1748. doi: 10.1002/art.42616. PMID: 37219936

[50] Takeuchi T Miyauchi E Kanaya T Kato T Nakanishi Y Watanabe T . Acetate differentially regulates IgA reactivity to commensal bacteria. Nature. (2021) 595(7868):560–564. doi: 10.1038/s41586-021-03727-5. PMID: 34262176

[51] Li H Limenitakis JP Greiff V Yilmaz B Schären O Urbaniak C . Mucosal or systemic microbiota exposures shape the B cell repertoire. Nature. (2020) 584(7820):274–278. doi: 10.1038/s41586-020-2564-6. PMID: 32760003

[52] Chriswell ME Lefferts AR Clay MR Chriswell ME Lefferts AR Clay MR . Clonal IgA and IgG autoantibodies from individuals at risk for rheumatoid arthritis identify an arthritogenic strain of *Subdoligranulum*. Sci Transl Med. (2022) 14(668):eabn5166. doi: 10.1126/scitranslmed.abn5166. PMID: 36288282 PMC9804515

[53] Takahashi D Hoshina N Kabumoto Y Maeda Y Suzuki A Tanabe H . Microbiota-derived butyrate limits the autoimmune response by promoting the differentiation of follicular regulatory T cells. EBioMedicine. (2020) 58:102913. doi: 10.1016/j.ebiom.2020.102913. PMID: 32711255 PMC7387783

[54] Zamani B Golkar HR Farshbaf S Emadi-Baygi M Tajabadi-Ebrahimi M Jafari P . Clinical and metabolic response to probiotic supplementation in patients with rheumatoid arthritis: a randomized, double-blind, placebo-controlled trial. Int J Rheum Dis. (2016) 19(9):869–879. doi: 10.1111/1756-185X.12888. PMID: 27135916

[55] Jubair WK Hendrickson JD Severs EL Schulz HM Adhikari S Ir D . Modulation of inflammatory arthritis in mice by gut microbiota through mucosal inflammation and autoantibody generation. Arthritis Rheumatol. (2018) 70(8):1220–1233. doi: 10.1002/art.40490. PMID: 29534332 PMC6105374

[56] Ollier WE MacGregor A . Genetic epidemiology of rheumatoid disease. Br Med Bull. (1995) 51(2):267–285. doi: 10.1093/oxfordjournals.bmb.a072960. PMID: 7552063

[57] Mangalea MR Paez-Espino D Kieft K . Individuals at risk for rheumatoid arthritis harbor differential intestinal bacteriophage communities with distinct metabolic potential. Cell Host Microbe. (2021) 29(5):726–739.e5. doi: 10.1016/j.chom.2021.03.020. PMID: 33957082 PMC8186507

[58] Wilson TM Trent B Kuhn KA Demoruelle MK . Microbial influences of mucosal immunity in rheumatoid arthritis. Curr Rheumatol Rep. (2020) 22(11):83. doi: 10.1007/s11926-020-00960-1. PMID: 33025188 PMC7876727

[59] Pérez-Pérez ME Nieto-Torres E Bollain-y-Goytia JJ Delgadillo-Ruíz L . Protein citrullination by peptidyl arginine deiminase/arginine deiminase homologs in members of the human microbiota and its recognition by anti-citrullinated protein antibodies. Int J Mol Sci. (2024) 25(10):5192. doi: 10.3390/ijms25105192. PMID: 38791230 PMC11121387

[60] Ma Y Wei X Peng J Wei F Wen Y Liu M . *Ephedra sinica* polysaccharide regulate the anti-inflammatory immunity of intestinal microecology and bacterial metabolites in rheumatoid arthritis. Front Pharmacol. (2024) 15:1414675. doi: 10.3389/fphar.2024.1414675. PMID: 38846095 PMC11153800

[61] Derksen VF Martinsson K van Mourik AG Wagenaar CA Toes RE Walrabenstein W . Evidence of site-specific mucosal autoantibody secretion in rheumatoid arthritis. Arthritis Rheumatol. (2025) 77(3):272–82. doi: 10.1002/art.43036. PMID: 39420623 PMC11865693

[62] Jhun J Min HK Ryu J Lee SY Ryu JG Choi JW . *Lactobacillus sakei* suppresses collagen-induced arthritis and modulates the differentiation of T helper 17 cells and regulatory B cells. J Transl Med. (2020) 18(1):317. doi: 10.1186/s12967-020-02477-8. PMID: 32799896 PMC7429687

[63] Duan H Wang L Huangfu M . The impact of microbiota-derived short-chain fatty acids on macrophage activities in disease: mechanisms and therapeutic potentials. Biomed Pharmacother. (2023) 165:115276. doi: 10.1016/j.biopha.2023.115276. PMID: 37542852

[64] Moen K Brun JG Valen M Skartveit L Eribe EK Olsen I . Synovial inflammation in active rheumatoid arthritis and psoriatic arthritis facilitates trapping of a variety of oral bacterial DNAs. Clin Exp Rheumatol. (2006) 24(6):656–663. 17207381

[65] Schott EM Farnsworth CW Grier A Lillis JA Soniwala S Dadourian GH . Targeting the gut microbiome to treat the osteoarthritis of obesity. JCI Insight. (2018) 3(8):e95997. doi: 10.1172/jci.insight.95997. PMID: 29669931 PMC5931133

[66] Moon J Lee AR Kim H Jhun J Lee SY Choi JW . *Faecalibacterium prausnitzii* alleviates inflammatory arthritis and regulates IL-17 production, short chain fatty acids, and the intestinal microbial flora in experimental mouse model for rheumatoid arthritis. Arthritis Res Ther. (2023) 25(1):130. doi: 10.1186/s13075-023-03118-3. PMID: 37496081 PMC10373287

[67] Van Raemdonck K Umar S Palasiewicz K Van Raemdonck K Umar S Palasiewicz K . CCL21/CCR7 signaling in macrophages promotes joint inflammation and Th17-mediated osteoclast formation in rheumatoid arthritis. Cell Mol Life Sci. (2020) 77(7):1387–1399. doi: 10.1007/s00018-019-03235-w. PMID: 31342120 PMC10040247

[68] Li B Ding M Chen C Zhao J Shi G Ross P . *Bifidobacterium longum* subsp. *infantis* B6MNI alleviates collagen-induced arthritis in rats via regulating 5-HIAA and Pim-1/JAK/STAT3 inflammation pathways. J Agric Food Chem. (2023) 71(46):17819–17832. doi: 10.1021/acs.jafc.3c05371. PMID: 37906736

[69] Naskar D Teng F Felix KM Bradley CP Wu HJJ . Synthetic retinoid AM80 ameliorates lung and arthritic autoimmune responses by inhibiting T follicular helper and Th17 cell responses. J Immunol. (2017) 198(5):1855–1864. doi: 10.4049/jimmunol.1601776. PMID: 28130500 PMC5324833

[70] May E Märker-Hermann E Wittig BM May E Märker-Hermann E Wittig BM . Identical T-cell expansions in the colon mucosa and the synovium of a patient with enterogenic spondyloarthropathy. Gastroenterology. (2000) 119(6):1745–1755. doi: 10.1053/gast.2000.20173. PMID: 11113096

[71] Pianta A Arvikar S Strle K Drouin EE Wang Q Costello CE . Evidence of the Immune Relevance of *Prevotella copri*, a Gut Microbe, in Patients With Rheumatoid Arthritis. Arthritis Rheumatol. (2017) 69(5):964–975. doi: 10.1002/art.40003. PMID: 27863183 PMC5406252

[72] Salmi M Andrew DP Butcher EC Jalkanen S . Dual binding capacity of mucosal immunoblasts to mucosal and synovial endothelium in humans: dissection of the molecular mechanisms. J Exp Med. (1995) 181(1):137–149. doi: 10.1084/jem.181.1.137. PMID: 7528765 PMC2191840

[73] Salmi M Rajala P Jalkanen S . Homing of mucosal leukocytes to joints. Distinct endothelial ligands in synovium mediate leukocyte-subtype specific adhesion. J Clin Invest. (1997) 99(9):2165–2172. doi: 10.1172/JCI119389. PMID: 9151788 PMC508046

[74] Padyukov L . Genetics of rheumatoid arthritis. Semin Immunopathol. (2022) 44(1):47–62. doi: 10.1007/s00281-022-00912-0. PMID: 35088123 PMC8837504

[75] Larid G Pancarte M Offer G Clavel C Martin M Pradel V . In rheumatoid arthritis patients, HLA-DRB1*04:01 and rheumatoid nodules are associated with ACPA to a particular fibrin epitope. Front Immunol. (2021) 12:692041. doi: 10.3389/fimmu.2021.692041. PMID: 34248985 PMC8264359

[76] Asquith M Sternes PR Costello ME Karstens L Diamond S Martin TM . HLA alleles associated with risk of ankylosing spondylitis and rheumatoid arthritis influence the gut microbiome. Arthritis Rheumatol. (2019) 71(10):1642–1650. doi: 10.1002/art.40917. PMID: 31038287

[77] Gomez A Luckey D Yeoman CJ Marietta EV Berg Miller ME Murray JA . Loss of sex and age driven differences in the gut microbiome characterize arthritis-susceptible 0401 mice but not arthritis-resistant 0402 mice. PLoS One. (2012) 7(4):e36095. doi: 10.1371/journal.pone.0036095. PMID: 22553482 PMC3338357

[78] Wells PM Adebayo AS Bowyer RC Freidin MB Finckh A Strowig T . Associations between gut microbiota and genetic risk for rheumatoid arthritis in the absence of disease: a cross-sectional study. Lancet Rheumatol. (2020) 2(7):e418–e427. doi: 10.1016/S2665-9913(20)30064-3. PMID: 33345197 PMC7729822

[79] Zhang X Chen BD Zhao LD Li H . The gut microbiota: emerging evidence in autoimmune diseases. Trends Mol Med. (2020) 26(9):862–873. doi: 10.1016/j.molmed.2020.04.001. PMID: 32402849

[80] Pianta A Arvikar SL Strle K Pianta A Arvikar SL Strle K . Evidence of the immune relevance of *Prevotella copri*, a gut microbe, in patients with rheumatoid arthritis. Arthritis Rheumatol. (2017) 69(5):964–975. doi: 10.1002/art.40003. PMID: 27863183 PMC5406252

[81] Pianta A Arvikar SL Strle K Pianta A Arvikar SL Strle K . Two rheumatoid arthritis-specific autoantigens correlate microbial immunity with autoimmune responses in joints. J Clin Invest. (2017) 127(8):2946–2956. doi: 10.1172/JCI93450. PMID: 28650341 PMC5531397

[82] Zhang X Zhang D Jia H . The oral and gut microbiomes are perturbed in rheumatoid arthritis and partly normalized after treatment. Nat Med. (2015) 21(8):895–905. doi: 10.1038/nm.3914. PMID: 26214836

[83] Liang B Ge C Lönnblom E . The autoantibody response to cyclic citrullinated collagen type II peptides in rheumatoid arthritis. Rheumatology (Oxford). (2019) 58(9):1623–33. doi: 10.1093/rheumatology/kez073. PMID: 30892636

[84] Yordanov M Tchorbanov A Ivanovska N . *Candida albicans* cell-wall fraction exacerbates collagen-induced arthritis in mice. Scand J Immunol. (2005) 61(4):301–308. doi: 10.1111/j.1365-3083.2005.01575.x. PMID: 15853911

[85] Costalonga M Hodges JS Herzberg MC . *Streptococcus sanguis* modulates type II collagen-induced arthritis in DBA/1J mice. J Immunol. (2002) 169(4):2189–2195. doi: 10.4049/jimmunol.169.4.2189. PMID: 12165549

[86] Suurmond J Diamond B . Autoantibodies in systemic autoimmune diseases: specificity and pathogenicity. J Clin Invest. (2015) 125(6):2194–2202. doi: 10.1172/JCI78084. PMID: 25938780 PMC4497746

[87] Poulsen TBG Damgaard D Jørgensen MM Senolt L Blackburn JM Nielsen CH . Identification of Novel Native Autoantigens in Rheumatoid Arthritis. Biomedicines. (2020) 8(6):141. doi: 10.3390/biomedicines8060141. PMID: 32486012 PMC7345460

[88] Yao Y Cai X Zheng Y Zhang M Fei W Sun D . Short-chain fatty acids regulate B cells differentiation via the FFA2 receptor to alleviate rheumatoid arthritis. Br J Pharmacol. (2022) 179(17):4315–4329. doi: 10.1111/bph.15852. PMID: 35393660

[89] Rosser EC Piper CJ Matei DE Blair PA Rendeiro AF Orford M . Microbiota-derived metabolites suppress arthritis by amplifying aryl-hydrocarbon receptor activation in regulatory B cells. Cell Metab. (2020) 31(4):837–851.e10. doi: 10.1016/j.cmet.2020.03.003. PMID: 32213346 PMC7156916

[90] He J Chu Y Li J Meng Q Liu Y Jin J . Intestinal butyrate-metabolizing species contribute to autoantibody production and bone erosion in rheumatoid arthritis. Sci Adv. (2022) 8(6):eabm1511. doi: 10.1126/sciadv.abm1511. PMID: 35148177 PMC11093108

[91] Corrêa-Oliveira R Fachi JL Vieira A Sato FT Vinolo MAR . Regulation of immune cell function by short-chain fatty acids. Clin Transl Immunology. (2016) 5(4):e73. doi: 10.1038/cti.2016.17. PMID: 27195116 PMC4855267

[92] Kibbie JJ Dillon SM Thompson TA . Butyrate directly decreases human gut lamina propria CD4 T cell function through histone deacetylase (HDAC) inhibition and GPR43 signaling. Immunobiology. (2021) 226(5):152126. doi: 10.1016/j.imbio.2021.152126. PMID: 34365090 PMC8478853

[93] McBride DA Dorn NC Yao M . Short-chain fatty acid-mediated epigenetic modulation of inflammatory T cells in vitro. Drug Deliv Transl Res. (2023) 13(7):1912–1924. doi: 10.1007/s13346-022-01284-6. PMID: 36566262 PMC10695156

[94] Zou F Qiu Y Huang Y . Effects of short-chain fatty acids in inhibiting HDAC and activating p38 MAPK are critical for promoting B10 cell generation and function. Cell Death Dis. (2021) 12(6):582. doi: 10.1038/s41419-021-03880-9. PMID: 34099635 PMC8184914

[95] Challa AA Lewandowski ED . Short-chain carbon sources: exploiting pleiotropic effects for heart failure therapy. JACC Basic Transl Sci. (2022) 7(7):730–742. doi: 10.1016/j.jacbts.2021.12.010. PMID: 35958686 PMC9357564

[96] Weyand CM Goronzy JJ . Metabolic checkpoints in rheumatoid arthritis. Semin Arthritis Rheum. (2025) 70S:152586. doi: 10.1016/j.semarthrit.2024.152586. PMID: 39550308 PMC11761375

[97] Zamani B Golkar HR Farshbaf S Emadi-Baygi M Tajabadi-Ebrahimi M Jafari P . Clinical and metabolic response to probiotic supplementation in patients with rheumatoid arthritis: a randomized, double-blind, placebo-controlled trial. Int J Rheum Dis. (2016) 19(9):869–879. doi: 10.1111/1756-185X.12888. PMID: 27135916

[98] Aqaeinezhad Rudbane SM Rahmdel S Abdollahzadeh SM Zare M Bazrafshan A Mazloomi SM . The efficacy of probiotic supplementation in rheumatoid arthritis: a meta-analysis of randomized, controlled trials. Inflammopharmacology. (2018) 26(1):67–76. doi: 10.1007/s10787-017-0436-y. PMID: 29302905

[99] Jeong Y Jhun J Lee SY Na HS Choi J Cho KH . Therapeutic potential of a novel *Bifidobacterium* identified through microbiome profiling of RA patients with different RF levels. Front Immunol. (2021) 12:736196. doi: 10.3389/fimmu.2021.736196. PMID: 34867956 PMC8634832

[100] Kim JE Chae CS Kim GC Hwang W Hwang JS Hwang SM . *Lactobacillus helveticus* suppresses experimental rheumatoid arthritis by reducing inflammatory T cell responses. J Funct Foods. (2015) 13:350–362. doi: 10.1016/j.jff.2015.01.002, PMID: 41909469

[101] Yamashita M Matsumoto K Endo T Ukibe K Hosoya T Matsubara Y . Preventive effect of *Lactobacillus helveticus* SBT2171 on collagen-induced arthritis in mice. Front Microbiol. (2017) 8:1159. doi: 10.3389/fmicb.2017.01159. PMID: 28680422 PMC5478730

[102] West AC Mizoro Y Wood SH Ince LM Iversen M Jørgensen EH . Immunologic profiling of the Atlantic Salmon gill by single nuclei transcriptomics. Front Immunol. (2021) 12:669889. doi: 10.3389/fimmu.2021.669889. PMID: 34017342 PMC8129531

[103] Schmidt CJ Wenndorf K Ebbers M Volzke J Müller M Strübing J . Infection with *Clostridioides difficile* attenuated collagen-induced arthritis in mice and involved mesenteric Treg and Th2 polarization. Front Immunol. (2020) 11:571049. doi: 10.3389/fimmu.2020.571049. PMID: 33193352 PMC7662472

[104] Surowiec I Ärlestig L Rantapää-Dahlqvist S Trygg J . Metabolite and lipid profiling of biobank plasma samples collected prior to onset of rheumatoid arthritis. PLoS One. (2016) 11(10):e0164196. doi: 10.1371/journal.pone.0164196. PMID: 27755546 PMC5068821

[105] Wang Q Zhang SX Chang MJ Qiao J Wang CH Li XF . Characteristics of the gut microbiome and its relationship with peripheral CD4^+^ T cell subpopulations and cytokines in rheumatoid arthritis. Front Microbiol. (2022) 13:799602. doi: 10.3389/fmicb.2022.799602. PMID: 35185845 PMC8851473

[106] Yanagisawa S Nagasaki K Chea C Ando T Ayuningtyas NF Inubushi T . Oral administration of bovine lactoferrin suppresses the progression of rheumatoid arthritis in an SKG mouse model. PLoS One. (2022) 17(2):e0263254. doi: 10.1371/journal.pone.0263254. PMID: 35148358 PMC8836292

[107] Ali EA Barakat BM Hassan R . Antioxidant and angiostatic effect of *Spirulina platensis* suspension in complete Freund's adjuvant-induced arthritis in rats. PLoS One. (2015) 10(4):e0121523. doi: 10.1371/journal.pone.0121523. PMID: 25853428 PMC4390336

[108] Yeom J Yim DJ Ma S . *Propionibacterium freudenreichii* inhibits RANKL-induced osteoclast differentiation and ameliorates rheumatoid arthritis in collagen-induced arthritis mice. Microorganisms. (2021) 10(1):48. doi: 10.3390/microorganisms10010048. PMID: 35056497 PMC8780394

[109] Qin Q Hu G Zhou X Zhu R Chen J Zeng K . Therapeutic potential of the probiotic *Lactiplantibacillus plantarum* BX62 and its postbiotics in alleviating rheumatoid arthritis in mice. Curr Res Food Sci. (2024) 9:100915. doi: 10.1016/j.crfs.2024.100915. PMID: 39582573 PMC11585737

[110] Philippou E Nikiphorou E . Are we really what we eat? Nutrition and its role in the onset of rheumatoid arthritis. Autoimmun Rev. (2018) 17(11):1074–1077. doi: 10.1016/j.autrev.2018.05.009. PMID: 30213695

[111] Sköldstam L Hagfors L Johansson G . An experimental study of a Mediterranean diet intervention for patients with rheumatoid arthritis. Ann Rheum Dis. (2003) 62(3):208–214. doi: 10.1136/ard.62.3.208. PMID: 12594104 PMC1754463

[112] Wang Z Yu Y Liao J Hu W Bian X Wu J . S-propargyl-cysteine remodels the gut microbiota to alleviate rheumatoid arthritis by regulating bile acid metabolism. Front Cell Infect Microbiol. (2021) 11:670593. doi: 10.3389/fcimb.2021.670593. PMID: 34422677 PMC8378902

[113] van den Bemt BJ Zwikker HE van den Ende CH . Medication adherence in patients with rheumatoid arthritis: a critical appraisal of the existing literature. Expert Rev Clin Immunol. (2012) 8(4):337–351. doi: 10.1586/eci.12.23. PMID: 22607180

[114] Fidder HH Singendonk MM van der Have M Oldenburg B van Oijen MG . Low rates of adherence for tumor necrosis factor-α inhibitors in Crohn's disease and rheumatoid arthritis: results of a systematic review. World J Gastroenterol. (2013) 19(27):4344–4350. doi: 10.3748/wjg.v19.i27.4344. PMID: 23885145 PMC3718902

[115] Chen S Hoang MH Hu D He Q Gao Y . Gut microbiota in early and established stages of rheumatoid arthritis: from pathogenesis to promising prevention and treatment. Ann Med. (2026) 58(1):2613484. doi: 10.1080/07853890.2026.2613484. PMID: 41574450 PMC12833903

[116] Luo Y Tong Y Wu L Niu H Li Y Su LC . Alteration of Gut Microbiota in Individuals at High-Risk for Rheumatoid Arthritis Associated With Disturbed Metabolome and the Initiation of Arthritis Through the Triggering of Mucosal Immunity Imbalance. Arthritis Rheumatol. (2023) 75(10):1736–1748. doi: 10.1002/art.42616. PMID: 37219936

